# Maternal and Sociodemographic Factors Influence the Consumption of Ultraprocessed and Minimally-Processed Foods in Pregnant Women

**DOI:** 10.1055/s-0040-1712996

**Published:** 2020-07

**Authors:** Monique Tavares Pereira, Monica Cattafesta, Edson Theodoro dos Santos Neto, Luciane Bresciani Salaroli

**Affiliations:** 1 Graduate Program in Nutrition and Health, Department of Integrated Health Education, Universidade Federal do Espírito Santo, Vitória, ES, Brazil; 2 Graduate Program in Collective Health, Universidade Federal do Espírito Santo, Vitória, ES, Brazil

**Keywords:** feeding behavior, dietary habits, ultraprocessed foods, pregnant, maternal nutrition, comportamento alimentar, hábitos alimentares, alimentos ultraprocessados, gestante, nutrição materna

## Abstract

**Objective** To analyze the consumption of minimally-processed and ultraprocessed foods in relation with sociodemographic variables, maternal habits, educational activity received during prenatal care and clinical history.

**Methods** A cross-sectional, analytical and descriptive study with 1,035 pregnant women who lives in the municipalities of the metropolitan region of Grande Vitória, Espírito Santo, Brazil (RMGV-ES), and who were hospitalized in establishments of the Unified Health System (SUS) due to childbirth (April–September 2010). The food frequency questionnaire, pregnant woman's card and information from the medical records of the health facility unit were analyzed. The Chi-square test and the binary logistic regression model were used to investigate the association between the independent variables and the consumption of ultraprocessed foods.

**Results** It was identified that pregnant women ≤19 years of age were 2.9 times more likely to consume ultraprocessed foods (confidence interval [CI] 95% 1.683–5.168, *p* < 0.001), while those ≥35 years old were less likely to consume them (odds ratio [OR] 0.265, 95% CI 0.105–0.666, *p* = 0.005). Maternal smoking increased the odds of consumption of ultraprocessed foods by 2.2 times (95% CI 1.202–4.199, *p* = 0.011) and pregnant women who did not obtain information on healthy food during prenatal care presented 54.1% less chances of consuming minimally-processed foods (OR 0.459, 95% CI 0.307–0.687, *p* < 0.001).

**Conclusion** Smoking during the gestational period and being a teenager are factors that influence the consumption of ultraprocessed foods of pregnant women. Race/color, head of household, age group, receiving of information about feeding in the prenatal period and not having smoked in gestation determined the consumption of minimally-processed foods.

## Resumo

**Objetivo** Analisar o consumo de alimentos minimamente processados e ultraprocessados e a sua associação com variáveis sociodemográficas, hábitos maternos, atividade educacional recebida durante o pré-natal e histórico clínico.

**Métodos** Estudo transversal, analítico e descritivo com 1.035 gestantes que moram nos municípios da Região Metropolitana da Grande Vitória, Espírito Santo, Brasil (RMGV-ES), e que foram internadas em estabelecimentos do Sistema Único de Saúde (SUS) devido ao parto (abril–setembro de 2010). Foram analisados o questionário de frequência alimentar, o cartão da gestante e as informações dos prontuários da unidade de saúde. O teste do Qui-quadrado e o modelo de regressão logística binária foram utilizados para investigar a associação entre as variáveis independentes e o hábito alimentar.

**Resultados** Identificou-se que as gestantes com idade ≤19 anos tinham 2,9 vezes mais chances de consumirem alimentos ultraprocessados (intervalo de confiança [IC] 95% 1,683–5,168; *p* < 0,001), enquanto aquelas com ≥ 35 anos tinham menos chances de consumí-los (razão de chances [RC] 0,265; IC 95% 0,105–0,666; *p* = 0,005). O tabagismo materno aumentou as chances de consumo de alimentos ultraprocessados em 2,2 vezes (IC95% 1,202–4,199; *p* = 0,011) e as gestantes que não receberam orientações sobre alimentação saudável durante o pré-natal apresentaram 54,1% menos chances de consumirem alimentos minimamente processados (RC 0,459; IC95% 0,307–0,687; *p* < 0,001).

**Conclusão** Fumar durante o período gestacional e ser adolescente são fatores que influenciam o consumo de alimentos ultraprocessados em gestantes. Raça/cor, chefe da família, faixa etária, recebimento de informações sobre alimentação no pré-natal e não fumar na gestação determinaram o consumo de minimamente processados.

## Introduction

Major changes have occurred in the dietary patterns of the population in recent years, mainly in relation to the substitution of nutrient-rich *in natura* foods for industrialized foods with high energy density and low nutritional quality.^1^ Factors such as the search for practicality and absence of time have led to various social changes and changes in health and food consumption of the population.^2^ Given the scenario of modification of food patterns and changes in the forms of food and beverage processing, Monteiro et al. (2016)^3^ proposed a new system for food classification. For this new classification, food items were grouped according to the extent and purpose of processing. Titled *NOVA*, consumption items are classified into four categories: *in natura* or minimally-processed foods; processed culinary ingredients; processed foods and ultraprocessed foods.^3^ The presence of ultraprocessed foods in the food consumption of Brazilians has been gradually expanding, with the need for a deep investigation of their impact on the health of the population.^3^ ^4^ Although there is a trend of high intake of ultraprocessed foods at all stages of the life cycle,^5^ ^6^ it is during gestation that women are more likely to change their pattern of food consumption due to concern about their maternal responsiveness.^7^ Thus, we analyzed the consumption of minimally-processed and ultraprocessed foods in relation with sociodemographic factors, maternal habits, educational activities and clinical history of pregnant women during prenatal care.

## Methods

This is a cross-sectional, analytical and descriptive study, with data collected from April to September 2010. The data of the present study are part of a larger study titled “Evaluation of the Quality of Prenatal Care in the Metropolitan Region of Grande Vitória, Espírito Santo, Brazil (RMGV-ES): Access and Integration of Health Services.”^8^ The sample consisted of pregnant women living in the municipalities of the metropolitan region of Grande Vitória, ES (MRGV-ES), hospitalized in establishments of the Unified Health System (SUS, in the Portuguese acronym) due to childbirth. This research was conducted in eight health facilities of the MRGV-ES, with these being contracted or belonging to the SUS. To calculate the sample size, we used the sample size formula to estimate a proportion of women studied. Through the data provided by the Information System on Live Births (SINASC, in the Portuguese acronym), a total of 17,980 live births were established in the public network or through the SUS, which number approximately reflects the number of pregnant women residing in the MRGV-ES. The indicator “Percentage of Live Births With by Seven or More Prenatal Care Consultations” was also considered, as an approximation of the utilization of health services during pregnancy. Considering a desired precision of 4%, drawing effect equal to 1.5 and significance level of 5%, calculations resulted in the sample size of 850 women. The total was increased by ∼ 30% to consider possible losses or refusals. A pilot study was performed with 67 parturients (not included in the study) in one of the establishments where the main study was conducted, for verification of the graphical presentations of the questionnaires, as well as the test on the understanding of their items and the evaluation of the average time of completion of the forms. All questions were tested by the Kappa, weighted Kappa and McNemar tests. These analyses demonstrated their applicability in the population studied, including questions about food consumption. The data were collected by a team through a closed structured interview, a full copy of the pregnant woman's card, and the retrieval of information from the medical records of the health facility where the delivery was performed. The interviewers visited at least once a week all eight maternity hospitals included in the study, approached women at the time after the birth, when they verified the possibility of the interview and possession of the pregnant woman's card. Women who did not have a pregnant woman's card were excluded, as well as those who underwent prenatal (all or part) follow-up in the private subsystem; pregnant women hospitalized with complications who did not have babies; and those who had follow-up in the municipalities outside the MRGV-ES. The women who had cesarean delivery had their data collected 12 hours after delivery, so that the anesthetic effects of the surgical procedure would not interfere with the responses. The race/color variable was determined by autoclassification as white, black, or brown (brown/mulatto). In relation to age group, these were categorized as “ ≤ 19 years,” “between 20 and 34 years,” or “ ≥ 35 years,” and the conjugal situation was categorized as “lives with partner” or “does not live with partner.” The variable maternal occupation was classified as “paid work” for pregnant women who had some work with remuneration, and “without paid work,” for those pregnant women who did not have paid work. In relation to the variable head of the family, these were categorized as “own,” “companion,” or “other.” Socioeconomic classification was determined based on the criterion of Brazilian Economic Classification of the Brazilian Association of Research Companies.^9^ The pregnant women were classified as class A/B, C1/C2, or D/E to improve the description of the outcomes. The use of tobacco and alcohol during pregnancy was analyzed through questions with direct answers (“yes” or “no”). It was questioned whether during the prenatal period there was information about the advantages of healthy food and its importance for the prevention of children's health problems. This variable was dichotomized in “yes” or “no.” The data on prenatal medical care were analyzed considering the number of prenatal consultations reported by the pregnant woman. This variable was classified as having performed from “one to five” or “six or more” prenatal visits. Fifteen food items registered through the questionnaire applied were evaluated. This questionnaire was based on the Vigilance System for Risk Factors for Non-Communicable Chronic Diseases of the Brazilian Ministry of Health (Vigitel, in the Portuguese acronym), which annually evaluates the food habits in Brazilian capitals.^10^ The frequency of reported intake was converted to daily frequency, so that all food items had the same unit. The daily frequency values were multiplied by the portion of each item consumed, thus revealing the number of servings consumed daily.^11^ After the calculation of the weight of the frequency of consumption of each item, the analyzed foods were inserted into groups established by the classification of foods based on the extent and purpose of their processing by *NOVA*.^3^ The foods were grouped according to the criteria proposed by Monteiro et al. (2016)^3^ in the *NOVA* classification, which considers the characteristics of the purpose and extent of the industrial processing they have undergone: in natura or minimaly-processed, processed culinary ingredients, processed foods and ultraprocessed foods. For this study, the foods that were classified as minimally-processed and ultraprocessed were used. The Chi-square test was used to analyze the differences in proportions. The significance level of α < 5% was adopted. The distribution of the consumption of minimally-processed and ultraprocessed foods was evaluated in quartiles. The binary logistic regression model was used to investigate the association between the independent variables and the consumption of minimally-processed foods and consumption of ultraprocessed foods. The variables included in the binary logistic regression analysis were selected according to the statistical significance in the Chi-square test (*p* < 0.2). The lowest quartile (Q1) and the highest quartile (Q4) were analyzed for the purpose of analyzing the extremes of consumption with the independent variables. All statistical analyzes were performed in the IBM SPSS Statistics for Windows, version 22.0 (IBM Corp., Armonk, NY, USA). This study was conducted according to the guidelines laid down in the Declaration of Helsinki, and all procedures involving human subjects were approved by Research Ethics Committee of the Health Sciences Center of Universidade Federal do Espírito Santo (grant number 3.060.797) and CAAE (grant number 99562818.0.0000.5060). Written informed consent was obtained from all subjects.

## Results

The sociodemographic variables, maternal habits, educational activity and clinical history were analyzed according to the quartiles of minimally-processed food consumption of 1,035 pregnant women (Table 1). The age group and the consumption of minimally-processed foods (*p* = 0.015) were associated, and it was observed that pregnant women aged ≤ 19 years had lower consumption of minimally-processed foods (32.8%, *n* = 76) than the other age groups. Women ≥ 35 years of age presented higher consumption of minimally-processed foods (32.5%, *n* = 26).

**Table 1:** Sociodemographic variables, maternal habits, educational ctivity and clinical history according to the consumption of minimally-processed foods by pregnant women

Variable	Minimally-processed food consumption*								P†	Total	
	Q1		Q2		Q3		Q4				
	n	%	n	%	n	%	n	%		n	%
Age, y (n 1,035)									0.015		
≤ 19	76	32.8	54	23.3	61	26.3	41	17.7		232	22.4
20–34	177	24.5	200	27.7	165	22.8	181	25.0		723	69.9
≥ 35	15	18.8	18	22.5	21	26.3	26	32.5		80	7.7
Race/color (n = 977)									0.096		
White	21	15.7	33	24.6	41	30.6	39	29.1		134	13.7
Brown	188	27.5	180	26.3	156	22.8	160	23.4		684	70.0
Black	41	25.8	43	27.0	36	22.6	39	24.5		159	16.3
Marital status (n = 1,030)									0.294		
Living with a partner	214	25.8	208	25.0	203	24.4	206	24.8		831	22.5
Not living with a partner	54	27.1	61	30.7	42	21.1	42	21.1		199	70.2
Maternal occupation (n = 1,034)									0.002		
No paid work	212	28.6	197	26.6	171	23.1	160	21.6		740	71.6
Paid work	55	18.7	75	25.5	76	25.9	88	29.9		294	28.4
Householder (n 1028)									0.073		
House owner	21	18.9	32	28.8	20	18.0	38	34.2		111	10.8
Partner's house	179	26.5	168	24.9	168	24.9	160	23.7		675	65.7
Others	65	26.9	70	28.9	57	23.6	50	20.7		242	23.5
Socioeconomic classification (n = 913)									0.994		
D/E	73	25.5	74	25.8	69	24.1	70	24.5		286	31.3
C1/C2	152	26.2	157	27.0	139	23.9	133	22.9		581	63.6
A/B	11	23.9	14	30.4	10	21.7	11	23.9		46	5.0
Information on prenatal diet (n = 1,012)									< 0.001		
No	161	32.7%	128	26.0%	98	19.9%	106	21.5%		493	48.7
Yes	94	18.1%	137	26.4%	147	28.3%	141	27.2%		519	51.3
Alcohol use during pregnancy (n = 1,034)									0.215		
No	238	25,5	239	25.6	229	24.5	228	24.4		934	90.3
Yes	30	30.0	32	32.0	18	18.0	20	20.0		100	9.7
Smoking during pregnancy (n = 1,026)									< 0.001		
No	211	23.5	241	26.9	212	23.7	232	25.9		896	87.3
Yes	53	40.8	29	22.3	33	25.4	15	11.5		130	12.7
Prenatal consultations (n = 948)									0.134		
≤ 5	83	34.2	67	27.5	61	27.1	59	25.0		270	28.5
> 6	160	65.8	177	72.5	164	72.9	177	75.0		678	71.5
Parity‡ (n 997)									0.991		
1	106	26.9	102	25.9	94	23.9	92	23.4		394	39.5
2	76	25.6	76	25.6	71	23.9	74	24.9		297	29.8
3 or more	74	24.2	80	26.1	75	24.5	77	25.2		306	30.7

Abbreviations: Q1, first quartile; Q2, second quartile; Q3, third quartile; Q4, fourth quartile.

* The distribution of the consumption of foods was evaluated in quartiles.

† The differences between quartiles were tested by the Chi-square test.

‡ Number of parturitions.

There was an association between maternal occupation (*p* = 0.002) and consumption of minimally-processed foods, in which it was possible to observe that pregnant women without paid work had a lower consumption of minimally-processed (28.6%, *n* = 212). On the other hand, women with paid work had a higher consumption of minimally-processed foods (29.9%, *n* = 88).

A statistical difference was identified in pregnant women who received information on healthy eating during prenatal care (*p* < 0.001) in relation to the consumption of minimally-processed foods, since women who did not receive information about food presented lower consumption of minimally-processed foods (32.7%, *n* = 161).

It was observed that pregnant women who smoked during pregnancy had lower consumption of minimally-processed foods (*p* < 0.001).

Regarding the analysis of sociodemographic variables, maternal habits, educational activity, and clinical history according to consumption of ultraprocessed foods (Table 2), there was a statistically significant association between age group (*p* < 0.001) and the consumption of ultraprocessed foods, with higher consumption of ultraprocessed foods among pregnant women age ≤ 19 years, and lower consumption of ultraprocessed foods among women in the age group of ≥ 35 years.

**Table 2:** Sociodemographic variables, maternal habits, educational activity and clinical history according to consumption of ultraprocessed foods by pregnant women

Variable	Ultraprocessed food consumption^∗^								*P†*	Total	
	Q1		Q2		Q3		Q4				
	n	%	n	%	n	%	n	%		n	%
Age, y (n = 1,035)									< 0.001		
≤ 19	36	15,5	55	23.7	59	25.4	82	35.3		232	22.4
20–34	199	27.5	185	25.6	173	23.9	166	23.0		723	69.9
≥ 35	32	40.0	27	33.8	15	18.8	6	7.5		80	7.7
Race/color (n = 977)									0.270		
White	38	28.4	34	25.4	30	22.4	32	23.9		134	13.7
Brown	182	26.6	167	24.4	168	24.6	167	24.4		684	70.0
Black	34	21.4	50	31.4	29	18.2	46	28.9		159	16.3
Marital status (n = 1,030)									0.631		
Living with a partner	209	25.2	219	26.4	201	24.2	202	24.3		831	80.7
Not living with a partner	56	28.1	45	22.6	46	23.1	52	26.1		199	19.3
Maternal occupation (n = 1,034)									0.023		
No paid work	177	23.9	190	25.7	174	23.5	199	26.9		740	71.6
Paid work	90	30.6	77	26.2	72	24.5	55	18.7		294	28.4
Householder (n = 1,028)									0.004		
Own	36	32.4	22	19.8	24	21.6	29	26.1		111	10.8
Companion	187	27.7	183	27.1	146	21.6	159	23.6		675	65.7
Others	41	16.9	62	25.6	73	30.2	66	27.3		242	23.5
Socioeconomic classification (n = 913)									0.695		
D/E	75	26.2	82	28.7	64	22.4	65	22.7		286	31.3
C1/C2	153	26.3	147	25.3	136	23.4	145	25.0		581	63.6
A/B	9	19.6	11	23.9	15	32.6	11	23.9		46	5.0
Information on prenatal diet (n = 1,012)									0.534		
No	135	27.4	133	27.0	114	23.1	111	22.5		493	48.7
Yes	128	24.7	132	25.4	125	24.1	134	25.8		519	51.3
Alcohol use during pregnancy (n = 1,034)									0.066		
No	248	26.6	247	26.4	216	23.1	223	23.9		934	90.3
Yes	19	19.0	20	20.0	31	31.0	30	30.0		100	9.7
Smoking during pregnancy (n = 1,026)									< 0.001		
No	246	27.5	235	26.2	215	24.0	200	22.3		896	87.3
Yes	19	14.6	31	23.8	31	23.8	49	37.7		130	12.7
Prenatal consultations (n = 948)									0.590		
≤ 5	67	27.0	68	27.0	69	32.1	66	28.3		270	28.5
> 6	181	73.0	184	73.0	146	67.9	167	71.7		678	71.5
Parity‡ (n 997)									0.161		
1	93	23.6	104	26.4	94	23.9	103	26.1		394	39.5
2	66	22.2	77	25.9	80	26.9	74	24.9		297	29.8
3 or more	96	31.3	77	25.1	64	20.9	69	22.5		306	30.7

Abbreviations: Q1, first quartile; Q2, second quartile; Q3, third quartile; Q4, fourth quartile.

* The distribution of the consumption of foods was evaluated in quartiles.

† The differences between quartiles were tested by the chi-square test.

‡ number of parturitions.

When evaluating the variables maternal occupation and head of the family, it was possible to identify an association with ultraprocessed food consumption, showing that women with paid work had lower ultraprocessed foods consumption, and women with unpaid work had higher consumption of ultraprocessed foods (*p* = 0.023). The pregnant women who were head of the family showed lower consumption of ultraprocessed foods (*p* = 0.004).

Women who smoked during pregnancy (37.7%, *n* = 49) had a statistically significant association with the highest quartile of ultraprocessed food consumption (*p* < 0.001).

Binary logistic regression analyses showed that pregnant women ≤ 19 years of age were 2.9 times more likely to consume ultraprocessed foods (odds ratio [OR] 2.950, confidence interval [CI] 95% 1.683–5.168, *p* < 0.001), while those ≥ 35 years of age had less chance to consume them (OR 0.265, 95% CI 0.105–0.666, *p* = 0.005). Smoking during pregnancy increased the chance of consumption of ultraprocessed foods by 2.2 times (OR 2.247, 95% CI 1.202–4.199, *p* = 0.011) (Table 3).

**Table 3:** Binary logistic regression analysis between consumption of ultraprocessed foods and associated variables in pregnant women

Variable	Ultraprocessed food consumption							
	Gross values				Adjusted values			
	P^a^	OR*	CI		P^a^	OR*	CI	
			LL 95%	UL 95%			LL 95%	UL 95%
Age, y								
20–34		1				1		
≤ 19	< 0.001	2.731	1.754	4.251	< 0.001	2.95	1.683	5.168
≥ 35	0.001	0.225	0.092	0.551	0.005	0.265	0.105	0.666
Maternal occupation								
No paid work		1				1		
Paid work	0.002	0.544	0.367	0.804	0.225	0.762	0.491	1.182
Householder								
Own		1				1		
Companion	0.842	1.056	0.62	1.798	0.98	0.992	0.543	1.814
Others	0.03	1.998	1.069	3.735	0.243	1.543	0.745	3.194
Alcohol use during pregnancy								
No		1				1		
Yes	0.067	1.756	0.9613	3.2074	0.298	1.480	0.708	3.092
Smoking during pregnancy								
No		1				1		
Yes	< 0.001	3.1721	1.809	5.5622	0.011	2.247	1.202	4.199
Parity								
1		1				1		
2	0.956	1.012	0.656	1.563	0.089	1.548	0.935	2.563
3 or more	0.042	0.649	0.427	0.985	0.562	1.170	0.689	1.987

Abbreviations: CI, confidence interval; LL, lower limit; OR, odds ratio; UL, upper limit.

a Statistical significance ≤ 0.2% in the Chi-square test were included in the analysis.

* ORs were adjusted to the other variables using binary logistic regression with 95%CIs.

As for the consumption of minimally-processed foods (Table 4), pregnant women aged ≤ 19 years were 45.3% less likely than pregnant women aged 20 to 34 to consume these foods (OR 0.547, 95% CI 0.324–0.923, *p* = 0.024). Brown-skinned women were 57.8% less likely to consume minimally-processed foods than white-skinned ones (OR 0.466, 95% CI 0.226–0.803, *p* = 0.008).

**Table 4:** Binary logistic regression analysis between consumption of minimally-processed foods and associated variables in pregnant women

Variable	Minimally-processed food consumption							
	Gross values				Adjusted values			
	*P* a	OR*	CI		*P* a	OR*	CI	
			LL 95%	UL 95%			LL 95%	UL 95%
Age, y								
20–34		1				1		
≤ 19	0.004	0.528	0.342	0.813	0.024	0.547	0.324	0.923
≥ 35	0.122	1.695	0.869	3.307	0.388	1.411	0.646	3.081
Race/color								
White		1				1		
Brown	0.007	0.458	0.259	0.811	0.008	0.426	0.226	0.803
Black	0.057	0.512	0.257	1.019	0.053	0.469	0.217	1.011
Marital status								
Living with a partner		1				1		
Not living with a partner	0.349	0.808	0.517	1.263	0.273	0.749	0.447	1.256
Maternal occupation								
No paid work		1				1		
Paid work	< 0.001	2.12	1.429	3.145	0.281	1.295	0.809	2.072
Householder								
Own		1				1		
Companion	0.016	0.494	0.278	0.877	0.017	0.432	0.217	0.859
Others	0.01	0.425	0.222	0.813	0.125	0.544	0.25	1.184
Information on prenatal feeding								
No		1				1		
Yes	< 0.001	2.278	1.593	3.259	< 0.001	2.177	1.455	3.257
Smoking during pregnancy								
No		1				1		
Yes	< 0.001	0.2574	0.1409	0.4703	< 0.001	0.269	0.132	0.545
Prenatal consultations								
<5		1				1		
>6	0.0287	1.5562	1.047	2.3132	0.1684	1.3771	0.8734	2.1713

Abbreviations: CI, confidence interval; LL, lower limit; OR, odds ratio; UL, upper limit.

a Statistical significance ≤ 0.2% in the Chi-square test were included in the analysis.

* ORs were adjusted to the other variables using binary logistic regression with 95%CIs.

Pregnant women who had the family headed by their partners were 56.8% less likely to consume minimally-processed foods than pregnant women who were the head of the family (OR 0.432, 95% CI 0.217–0.859, *p* = 0.017). However, women who received information on healthy food during prenatal care presented 2.17 times more chances to consume minimally-processed foods (OR 2.177, 95% CI 1.455–3.257, *p* = 0.001).

## Discussion

The present study allowed identifying the factors associated with the consumption of ultraprocessed and minimally-processed foods. The data show that the consumption of ultraprocessed products diverged between the age groups, demonstrating a lower consumption of ultraprocessed foods among older women. This finding was similar to that in the study by Alves-Santos et al.^12^ in which older women showed a lower intake of ultraprocessed foods than the younger ones, indicating the age factor as strongly associated with the consumption of minimally-processed foods. It is still consistent with the findings by McGowan and McAuliffe,^13^ in which older women tend to adhere to a “healthy conscience” pattern, consisting mainly of higher intakes of whole-grain breads, fruits, vegetables, low-fat milk, and white meat, among others. This relationship can also be observed in Brazilian households, considering the 2008 to 2009 family budgets survey, conducted with individuals who were 10 years of age or older, which showed the consumption of ultraprocessed products tends to decrease as age increases.^14^

However, younger pregnant women presented high consumption of ultraprocessed foods, in agreement with the results already presented in the adolescent population of the city of Rio de Janeiro, in which pregnant women presented a high frequency of consumption of soft drinks, pizza, potato chips, and salty snacks, indicating food habit that is harmful to health.^15^ The association between high consumption of ultraprocessed products and age groups can also be found in non-pregnant women, in which adolescents consumed and spent more to purchase ultraprocessed foods, compromising the quality of their diet.^16^

The present study showed that maternal habits may interfere with food consumption. Women who reported having smoked during pregnancy were associated with higher consumption of ultraprocessed foods. Knowing that maternal smoking has a negative impact on the health of the mother and is related to a series of risk behaviors because it is associated with a greater chance of maternal and fetal intercurrences,^17^ ^18^ it is important to offer pregnant women information about the effects of substances, which, in turn, will contribute to benefit obstetric outcomes.^18^ The impacts of maternal smoking on pregnancy outcomes are well documented and include predisposition to gestational diabetes,^19^ higher mortality and morbidity,^20^ and increased risk of preterm birth, and the consequences of cigarette smoking during pregnancy may accompany the baby throughout life.^21^ In addition, fetuses exposed to cigarette smoke during pregnancy tend to have a higher body mass index (BMI) in adulthood.^22^

Other adverse outcomes associated with maternal smoking include congenital malformations,^23^ ^24^ low birth weight^21^ ^22^ and restriction of intrauterine growth,^20^ ^25^ suggesting that any newborn with significant exposure to smoking during gestation is likely to be adversely affected to some degree due to the chemical and toxic additives of tobacco.^26^ The present study has shown that pregnant women who presented a maternal habit of not smoking during the gestational period had a better habit of food consumption. In light of that, women who quit smoking before or during pregnancy can substantially reduce risks for themselves and their children.^27^

Sociodemographic factors were associated with the consumption of minimally-processed foods, specifically race/skin color and being head of household. In a study of American women who were in the third trimester of pregnancy, it was shown that Black women tended to have higher caloric intake, consuming foods rich in fats, carbohydrates, and sugar and poor in important nutrients, such as folate and fiber.^28^

The head of household association and the consumption of healthier foods may demonstrate social inequalities, since female-headed households are largely associated with situations of economic vulnerability, in which women often end up in domestic activities and care of the offspring, which, in turn, results in greater difficulties to guarantee the subsistence of their own family, already with part-time affected generates dependency in low-paid jobs.^29^

Receiving information about healthy food during prenatal care was highlighted among women who had the highest consumption of minimally-processed foods, as recommended in the pregnant woman's booklet, which states that to have a healthy pregnancy the pregnant woman should seek to have a diet rich in natural foods and poor in processed foods, in favor of maternal well-being, growth, and adequate training of the fetus.^30^

Participants who did not receive information about healthy eating during the prenatal period had less chance of having healthier eating habits. Considering that such educational activities depend on health professionals, the challenge seems to be to understand the reasons why these behaviors are not being fully available to the target population.^31^ The educator role played by the health professional during prenatal consultations is essential, since pregnant women should be instructed to adopt improvements or maintenance of healthy eating practices, making changes and/or creating new habits and, thus promoting a pregnancy free of future intercurrences.^32^ With these data, prenatal monitoring is identified as an important source of information that may contribute to excellent results in food consumption during pregnancy.

In the study done by Barros et al.^15^ it was verified that the adolescent pregnant women who received information about healthy eating during the prenatal period had better results in nutrient consumption. Verbeke and De Bourdeaudhuij^33^ analyzed the eating behavior and nutritional choices of 148 pregnant women and 130 non-pregnant women and observed that pregnant women who followed nutritional recommendations made better eating choices than non-pregnant women. This corroborates the importance of prenatal follow-up, recognized as a relevant stage in the collection and perception of information, effectively contributing to improvements in the results of food consumption and gestation.^15^

The limitations of the present study are related to the possibility of occurrence of recall bias, because the data was obtained through an interview, but this limitation may have been minimized by investigating the pregnant woman's medical chart and/or card. Still, the method used to collect data on eating habits may have involved underestimation; however, it made it possible to assess the consumption of the main food items evaluated in national surveys. Nevertheless, despite the limitations pointed out, the data presented here reflect the reality of the public and/or contracted establishments that performed the deliveries by the SUS of the MRGV-ES, and the design adopted enables assuming that the conclusions can be used in similar contexts.

It is also highlighted that it is the first study to investigate the association between maternal variables and educational activity received during prenatal care with ultraprocessed and minimally-processed consumption and, differently from other studies that analyzed isolated foods, in this study we used a classification that groups the foods according to purpose and extent of processing.^3^

Although this classification was recent and in the present study the fourth quartile was used to define the high consumption of a certain group, it allowed to classify in a satisfactory way those foods that belonged to the group that presented high amounts of sugars, saturated fat, trans fat, and low amount of fiber, characteristics that the literature shows belong to the ultraprocessed foods, which intensifies the risks for various diseases.^1^ ^3^ ^34^ ^35^ ^36^

## Conclusion

The results of the present study suggest that being a pregnant adolescent and having smoked during pregnancy influence the increase in the consumption of ultraprocessed foods, which, consequently, can have an impact on the quality of the food choices of pregnant women.

The data also indicate that being an older pregnant woman, not being of brown skin color, being the head of the household, not having smoked cigarettes during pregnancy and having received information about healthy eating during prenatal care contribute to the better consumption of minimally-processed or *in natura* foods, thus offering better eating habits. It is necessary to evaluate the food consumption through these food groups, as it allows to identify the vulnerability of the population to food excesses, and thus to adapt and propose intervention measures that guarantee maternal and baby health. The present results point out that there is a need for the implementation of intervention measures in health service establishments with the objective of providing educational information and promoting healthy eating for mothers.
